# PRESIDENTIAL SYMPOSIUM: BUILDING AND LEVERAGING COMMUNITY PARTNERSHIPS THAT EMBRACE DIVERSITY, ENRICH DISCOVERY, AND REIMAGINE AGING

**DOI:** 10.1093/geroni/igac059.1460

**Published:** 2022-12-20

**Authors:** Kalisha Bonds Johnson, Brianna Morgan, Katherine Abbott

## Abstract

Are you an ESPO member or early careerist who is interested in creating meaningful and engaging partnerships with different stakeholders? Want to learn how to leverage partnerships across diverse research settings and make impactful change? Or are you unsure of all the “hype” surrounding engaging communities and key stakeholders? If you answered “yes” to any of these questions, this symposium is for you. The ESPO Presidential Symposium will highlight three speakers who will share their career stories and a discussant with expertise in building and keeping important collaborations that influence aging research. Speakers include Drs. Christopher G. Engeland, Andrea Gilmore-Bykovskyi, and Martina Roes. Dr. Katherine Abbott will serve as the discussant. Starting at the basic science end of the research spectrum, Dr. Engeland will focus on communicating and collaborating at the intersections between research settings to carry science from the bench to the bedside. Next, moving to research that focuses on aging older adults in clinical research, Dr. Gilmore-Bykovskyi will discuss how she has established, maintained, and evolved her career in Alzheimer’s disease and related dementias across diverse research paradigms. Lastly, Dr. Roes will provide and international perspective and shares how she builds trust with the co-creation of research with persons living with dementia and their significant others.

